# Free radical scavenging activities of **Cnidium officinale** Makino and **Ligusticum chuanxiong** Hort. methanolic extracts

**DOI:** 10.4103/0973-1296.71794

**Published:** 2010

**Authors:** Mahesh Ramalingam, Park Yong-Ki

**Affiliations:** *Oriental Medicine Research Institute, Dongguk University, 707, Seokjangdong, Gyeongju, Gyeongbuk 780-714, Republic of Korea*; 1 *Department of Herbology, College of Oriental Medicine, Dongguk University, 707, Seokjangdong, Gyeongju, Gyeongbuk 780-714, Republic of Korea*

**Keywords:** Antioxidant, Cnidium officinale, free radical scavenging, Ligusticum chuanxiong, Umbelliferae

## Abstract

**Background:**

Antioxidants from natural resources possess multifaceted and importance of the activities provides substantial scope in neurodegenerative diseases. The aim of this study was to assess and compare the free radical scavenging activities of **Cnidium officinale** and **Ligusticum chuanxiong**, which are closely related species.

**Materials and Methods:**

The scavenging activities of plant materials were evaluated using Trolox equivalent antioxidant capacity (TEAC), oxygen radical absorbance capacity (ORAC) and 1,1-diphenyl-2-picrylhydrazyl (DPPH), superoxide radical (O_2_·^-^), hydrogen peroxide (H_2_O_2_), hydroxyl (OH·), nitric oxide radical (NO·) and metal chelation. In addition, the cell viability and nitric oxide release were assayed using Neuro-2a (N2a) cells.

**Results:**

The methanolic extracts of **C. officinale** and **L. chuanxiong** showed scavenging activities of free radicals with an additional antioxidant capacity. Moreover, the efficacy on the cell viability and nitric oxide release in cell culture model has been established.

**Conclusion:**

Results of the present study suggests that the extracts of **C. officinale** and **L. chuanxiong** have comparatively similar free radical scavenging activities *in vitro* and may have important health effects.

## INTRODUCTION

Oxidative stress is a process defined as the condition occurring when the physiological balance between oxidants and antioxidants is disrupted in favor of the former with potential damage for the organism. Excessive reactive oxygen species (ROS), produced *in vivo* during oxidative reactions, are involved in the development of diseases.[1] In recent years, there has been a growing interest in finding natural antioxidants in plants because they inhibit oxidative damage and may consequently prevent inflammatory conditions, aging and neurodegenerative diseases.[2,3] Such natural antioxidants are believed to play a potential role in interfering with the oxidation process by reacting with free radicals, chelating catalytic metals and scavenging oxygen in biological systems.

**Cnidium officinale** Makino is a perennial herb native to China and is extensively cultivated in Korea, China and Japan. The rhizomes of *C. officinale* (Cinidii Rhizoma), which belongs to the Umbelliferae family, have been used as traditional oriental medicine in Korea. It has been shown that the dried rhizomes of *C. officinale* are used in the treatment of pain, inflammation, menstrual disturbance, and anti-vitamin deficiency disease, and also act as a blood pressure depressant. In addition, there are several reports suggesting that they have pharmacological properties to tumor metastasis and angiogenesis and that they act as an inhibitor of high glucose-induced proliferation of glomerular mesangial cells.[4,5] Rhizoma Chuanxiong, the dried rhizome of **Ligusticum chuanxiong** Hort (Umbelliferae), is one of the most commonly prescribed traditional Chinese medicinal herbs for the treatment of cerebro and cardio-vascular diseases.[6] It exhibits skin regeneration effects in patients with eczema and psoriasis, cardiovascular, antiplatelet, anti-inflammatory, and also antimicrobial and insecticidal effects.[7–11]

In folk medicine, the dried rhizomes of **C. officinale** and **L. chuanxiong** are widely used for amenorrhea, dysmenorrheal, pricking pain in the chest and hypochondriac regions, and furthermore, for dispelling wind and relieving pain from headache and rheumatic arthralgia.[12] Some composite components from these plants are butylphthalide, sedanonic acid, cnidilide, ligustilide, neocnidilide, etc.[13,14] Molecular results suggested that the origin of *L. chuanxiong* is *C. officinale*.[15] According to Lee *et al*.,[16] **C. officinale** and **L. chuanxiong** are closely related species with 98% of sequence identity. Previous studies from our laboratory showed that both **C. officinale** and **L. chuanxiong** had the same inhibitory activities on lipid peroxidation and there were no significant differences between two herbs. When the effects on xanthine oxidase and aldehyde oxidase activities were examined, *C. officinale* showed higher activity than that of *L. chuanxiong*.[17] Moreover, **C. officinale** and **L. chuanxiong** have been reported to have vasodilation effects.[18] Recently, Jeong *et al*.[14] reported that **C. officinale** and **L. chuanxiong** have potential roles in the chemoprevention of DNA damage and apoptosis induced by ultraviolet B and reduce the content or impact of ROS.

However, little has been known about the antioxidant roles of the methanol extracts from **C. officinale** and **L. chuanxiong** on apoptosis caused by ROS. Therefore, the objective of this study was to evaluate and compare their free radical scavenging activities *in vitro*. In this study, the antioxidant properties of methanol extracts from the rhizomes of **C. officinale** and **L. chuanxiong** were evaluated for Trolox equivalent antioxidant capacity (TEAC), oxygen radical absorbance capacity (ORAC), 1,1-diphenyl-2-picrylhydrazyl (DPPH) radical scavenging, superoxide radical (O_2_^·–^) scavenging, hydrogen peroxide (H_2_O_2_) scavenging, hydroxyl (OH^·^) radical scavenging, nitric oxide radical (NO^·^) scavenging and ferrous ion chelating efficacies. Moreover, the effects of **C. officinale** and **L. chuanxiong** on cell viability and nitric oxide (NO) release in Neuro-2a (N2a) cells were analyzed.

## MATERIALS AND METHODS

### Chemicals

DPPH, nitroblue tetrazolium salt (NBT), phenazine methosulfate (PMS), thiobarbituric acid (TBA), trichloroacetic acid (TCA), dimethyl sulfoxide (DMSO), sodium nitroprusside (SNP), sulfanilic acid, N-(1-naphthyl) ethylenediamine dihydrochloride, ethylenediaminetetraacetic acid (EDTA), FeSO_4_·7H_2_O, deoxyribose, 2,2’-azino-bis(3-ethylbenzthioziozline-6-sulfonic acid (ABTS), 2,2’-azobis (2-amidinopropane) dihydrochloride (AAPH), Trolox (6-hydroxy-2,5,7,8-tetramethylchroman-2-carboxylic acid), a water-soluble analog of vitamin E, and 3-(4,5-dimethyl-2-yl)-2,5-diphenyltetrazolium bromide (MTT) were purchased from Sigma-Aldrich Co. (St. Louis, MO, USA). All other chemicals used were of analytical grade, supplied by Fluka (Buchs, Switzerland) or Sigma-Aldrich Co.

### Plant materials and preparation of extracts

The roots of **C. officinale** and **L. chuanxiong** were collected in Yeong-Chun Province, Republic of Korea, during October 2007 and identified by Dr. YK Park, Department of Herbology, College of Oriental Medicine, Dongguk University (DUCOM), Republic of Korea. The voucher specimens (CR-K0710 and CR-J0710) were deposited in the Herbarium of DUCOM. Air-dried roots (200 g) were cut into small pieces and extracted with 1 l of 80% methanol in a reflux condenser for 3 h. The methanol extracts were filtered through Whatman No. 1 filter paper and concentrated in a vacuum evaporator (yields of 16.7%).

### Trolox equivalent antioxidant capacity assay

The antiradical properties of the extracts were determined using the TEAC assay. The TEAC assay is based on the reduction of the ABTS radical cation by antioxidants and was adapted with minor modifications.[19,20] ABTS radical cation was prepared by mixing ABTS stock solution (7 mM in water) with 2.45 mM potassium persulfate (K_2_S_2_O_8_). This mixture was left for 12–24 h in the dark until the reaction was complete and the absorbance was stable [Abs_734nm_ to 0.700 (±0.030)]. Prior to use in the assay, the ABTS^·+^ solution was diluted with phosphate buffered saline (PBS) to an absorbance of 0.70 (±0.02) at 734 nm and equilibrated at 30°C. Extract, ascorbic acid (as control) and Trolox (as standard) (20 µl) were dissolved in PBS, and then added to the ABTS^·+^ solution (1980 µl). The absorbance reading was taken exactly 6 min after initial mixing. Appropriate solvent blanks were run in each assay. The antioxidant capacity of the samples was calculated by determining the decrease in absorbance at different concentrations (0–150 µg/ml).

### Oxygen radical absorbance capacity assay

The ORAC assay was based upon a previous procedure described by Cao *et al*.[21] The reaction was carried out in 75 mM phosphate buffer (pH 7.0), and the final reaction mixture was 200 µl. Antioxidant (20 µl) and β-phycoerythrin (120 µl; 16.7 nM final concentration) solutions were placed in the well of the microplate. The mixture was preincubated for 15 min at 37°C. AAPH solution (60 µl; 32 mM final concentration) was added rapidly using a multichannel pipette. The microplate was immediately placed in the reader and the fluorescence recorded every minute for 80 min. The microplate was automatically shaken prior to each reading. A blank using phosphate buffer instead of the antioxidant solution and five calibration solutions using Trolox as antioxidant were also carried out in each assay. All the reaction mixtures were prepared in duplicate, and at least three independent assays were performed for each sample.

The final ORAC values were calculated by using a regression equation between the Trolox concentration and the net area under the fluorescein decay curve and were expressed as Trolox equivalents with mM/g. The normalized area under curve (AUC) was calculated as:

$$\mathrm{AUC}\;=\;(f_{0}\;+\;f_{1}\;+\;f_{2}\;+...\;+\;f_{n})/f_{0,}$$

where *f*_0_ is the initial fluorescence reading at 0 min and *f_k_* is the fluorescence reading at time *k* and *n* is the total number of time steps.

The AUC_net_ was obtained by subtracting the AUC of the blank from that of the sample or standard:

$${\mathrm{AUC}}_{\mathrm{net}}\;=\;{\mathrm{AUC}}_{\mathrm{sample}/\mathrm{standard}}\;-{\mathrm{AUC}}_{\mathrm{blank}}.$$

### 1,1-Diphenyl-2-picrylhydrazyl assay

The ability of the extract to scavenge DPPH radicals was assessed as described by Gyamfi *et al*.[22] It is one of the most extensively used antioxidant assays for plant samples. This method is based on scavenging of the DPPH radicals by the antioxidants, which produces a decrease in absorbance at 517 nm. When a solution of DPPH is mixed with a substance that can donate a hydrogen atom, the reduced form of the radical is generated, accompanied by loss of color. This delocalization is also responsible for the deep violet color, characterized by an absorption band in ethanol solution at about 517 nm. A 50-µl of aliquot of extract or control was mixed with 450 µl PBS (10 mM/l, pH 7.4) and 1.0 ml of methanolic DPPH (0.1 mM/l) solution. After a-30 min reaction, the absorbance was recorded at 517 nm.

### Superoxide radical scavenging assay

The scavenging activity against chemically generated superoxide radicals (O2^·–^) of the crude extracts was measured by means of spectrophotometric measurement of the product on reduction of NBT.[23] Superoxide anions were generated in a nonenzymatic PMS/NADH system. The reaction mixture contained 1 ml of test solution, 1.9 ml of 0.1 M phosphate buffer (pH 7.4), 1 ml of 20 µM PMS, 156 µM NADH, and 25 µM NBT in phosphate buffer (pH 7.4). After 2 min of incubation at 25°C, the color was read on a spectrophotometer at 560 nm against blank samples that contained no PMS.

### Hydrogen peroxide decomposition assay

H_2_O_2_ decomposition was determined according to the standard method.[24] The assay mixture contained 4 ml of H_2_O_2_ solution (80 mM) and 5 ml of phosphate buffer. One milliliter of the extracts was rapidly mixed with the reaction mixture by a gentle swirling motion. The reaction was run at room temperature. Then, 1 ml portion of the reaction mixture was blown into 2 ml of dichromate/acetic acid reagent at 60-second intervals. The decomposition of H_2_O_2_ was determined based on the standard plot for H_2_O_2_.[25]

### Hydroxyl radical scavenging assay

The ability of different extracts to scavenge the hydroxyl radicals (OH^·^) generated by the Fenton reaction was measured according to the modified method of Chung *et al*.[26] The Fenton reaction mixture containing 200 µl of 10 mM FeSO_4_·7H_2_O, 200 µl of 10 mM EDTA and 200 µl of 10 mM 2-deoxyribose was mixed with 1.2 ml of 0.1 M phosphate buffer (pH 7.4) containing 200 µl of extracts. Thereafter, 200 µl of 10 mM H_2_O_2_ was added to the mixture before incubation for 4 h at 37°C. Later, 1 ml of 2.8% TCA and 1 ml of 1% TBA were added and placed in a boiling water bath for 10 min. Then, the resultant mixture was allowed to cool up to room temperature and centrifuged at 395 ×g for 5 min. Absorbance was recorded at 532 nm using a UV-VIS spectrophotometer.

### Nitric oxide radical scavenging assay

SNP in methanol solution at physiological pH spontaneously produces nitric oxide (NO), which reacts with oxygen to produce nitrite ions, which can be determined by the use of the Griess Illosvoy reaction.[27] Griess Illosvoy reagent was slightly modified using naphthylethylenediamine dihydrochloride (0.1 w/v) instead of 1-naphthylamine (5%). Scavengers of nitric oxide compete with oxygen and reduce the production of nitric oxide.[28] The reaction mixture (3 ml) containing 2 ml of 10 mM SNP, 0.5 ml of PBS (pH 7.4, 0.01 M) and 0.5 ml of extract was incubated for 150 min at 25°C. Thereafter, 0.5 ml of the reaction mixture containing nitrite was pipetted and mixed with 1 ml of sulfanilic acid reagent (0.33% in 20% glacial acetic acid) and allowed to stand for 5 min for completing diazotization. Then, 1 ml of naphthylethylenediamine dihydrochloride (0.1%) was added and allowed to stand for 30 min in diffused light. The absorbance of the pink colored chromospheres was measured at 540 nm against the corresponding blank solutions in a 96-well plate, using ELISA reader.

### Ferrous ion chelating ability

The method of Decker and Welch was used to investigate the ferrous ion chelating ability of extracts.[29] A 5-ml amount of extract was mixed with 0.1 ml of 2 mM FeCl_2_ and 0.2 ml of 5 mM ferrozine solutions. The absorbance at 562 nm was determined after reaction for 10 min. A complex of Fe^2+^/ferrozine showed strong absorbance at 562 nm.

### Calculation of 50% inhibition concentration

All experiments were conducted in triplicate (*n* = 3) ± standard deviation (SD). The concentration of the extract that was required to scavenge 50% of radicals (IC_50_) was calculated by using the percent scavenging activities of five different extract concentrations. Percent scavenging activity was calculated as $\left[1\;-\;(\mathrm{Ai}\;-\;\mathrm{Aj})/\mathrm{Ac}\right]\;\times \;100,$

where *Ai* is the absorbance measured with the extract in the particular assay with an ROS source, *Aj* is the absorbance measured with the extract in the particular assay but without an ROS source and *Ac* is the absorbance of control with a particular solvent.

### Cell culture and treatments

N2a (CCL-131, American Type Culture Collection, Manassas, VA, USA) neuroblastoma cells were grown in Dulbecco’s modified Eagle’s medium (DMEM) supplemented with 10% heat inactivated fetal bovine serum (FBS) and 1% penicillin-streptomycin (Pen-Strep, GIBCO, Grand Island, NY, USA). Cultures were maintained in plastic tissue culture vessels in a humidified atmosphere at 37°C with 5% CO_2_. Cells were seeded at 5 × 10^4^/ml for cell viability and nitric oxide release estimation experiment and allowed to grow for 24 h. All the treatments were performed after 24 h duration in serum-free DMEM media. For treatments, serum-starved cells were incubated with **C. officinale** and **L. chuanxiong** (0–500 µg/ml) for 1 h.

### Determination of cell viability

Cell viability was measured by the MTT method. The assay is based on the ability of living cells to convert dissolved MTT into insoluble formazan by mitochondrial dehydrogenases in viable cells. The amount of formazan produced is proportional to the number of living cells. After treatments, cells were incubated with 0.5 mg/ml of MTT solution. Following additional 3 hours incubation at 37°C, the medium was removed and 100 µl of DMSO was added to dissolve the formazan crystals. The absorbance was read at 570 nm using a microplate reader. The optical density of the formazan formed in the control cells was taken as 100% viability. Data are mean percentages of viable cells versus the respective controls.

### Estimation of nitric oxide release

After treatments, the cells were centrifuged at 1000 ×g for 10 min and the supernatants were collected. The NO production was measured as the nitrite (NO_2_^–^) concentration according to the method of Green *et al*.[30] Supernatants (50 µl) were mixed with 100 µl of 0.1% sulfanilamide and 100 µl 0.1% *N* -1-naphthylethylenediamine dihydrochloride in 2.5% polyphosphoric acid. The absorbance was measured at 540 nm with a microplate reader. Sodium nitrate was used as a standard.

### Statistical analysis

All the data analysis was completed using the Graphpad PRISM 5.0 software. Data are expressed as mean ± SD. The significance level of treatment effects was determined using one way analysis of variance (ANOVA) followed by Tukey’s *post hoc* analysis and *P* values lower than 0.05 were considered statistically significant. All the experiments were performed for a minimum of three times.

## RESULTS AND DISCUSSION

Several pathological events, such as the inflammation process, coronary arterial disease, and aging phenomena, are associated with the generation of ROS. Thus, in this study, we demonstrated that extracts of **C. officinale** and **L. chuanxiong** also possess antioxidant properties, as they were able to protect the cells from oxidative damage and also inhibit the generation of ROS.

For TEAC assay, Table 1 shows the antiradical capacity of **C. officinale** and **L. chuanxiong** in an aqueous system, measured by assaying the ABTS radicals. The ABTS assay has been used to measure the total antioxidant activity in plant materials. As used by Rice-Evans and Miller,[31] TEAC reflects the relative ability of hydrogen or electron-donating antioxidants to scavenge the ABTS radical cation compared with that of Trolox. In this study, **C. officinale** and **L. chuanxiong** in the range of 0–150 µg/ml displayed antiradical activity, and the antiradical activity of these samples increased with increasing concentration of the extracts, indicating that **C. officinale** and **L. chuanxiong** showed scavenging activity of free radicals. As can be seen in Table 1, the scavenging ability of **C. officinale** and **L. chuanxiong** on ABTS radical cation was compared to that of ascorbic acid. In addition, *C. officinale* showed marked scavenging effect on ABTS cation radicals compared with that of the standard, ascorbic acid, determined through TEAC.

**Table 1:** Antioxidant activities of methanol extracts of **C. officinale** and **L. chuanxiong**

	TEAC (mM TE/g)	ORAC (mM TE/g)	DPPH (mM TE/g)
CO	2.022 ± 0.538	0.484 ± 0.162	2.942 ± 0.495
LC	1.249 ± 0.224	0.260 ± 0.015	4.658 ± 1.183
AA^a^	0.828 ± 0.010	0.030 ± 0.018	1.332 ± 0.027

Values are means ± SD of three measurements. CO: *C. officinale*, LC: *L. chuanxiong*, AA: Ascorbic acid, TEAC: Trolox equivalent antioxidant capacity, ORAC: Oxygen radical absorbance capacity, DPPH: 1,1-diphenyl-2-picrylhydrazyl

a positive control

Also, Table 1 shows the ORAC of samples. The highly fluorescent protein, beta-phycoerythrin (PE), derived from numerous species of red algae, has been used as the target of free radical damage.[32] Peroxyl radicals generated by the thermal decomposition of AAPH quench the fluorescence of phycoerythrin, whereas addition of an antioxidant that reacts rapidly with peroxyl radicals inhibits the loss of fluorescence intensity and this inhibition is proportional to the antioxidant activity. Final results can be calculated using the differences in areas under the phycoerythrin decay curves between the blank and a sample and are expressed in Trolox equivalents.[32] In this study, the results showed that **C. officinale** and **L. chuanxiong** exhibit antioxidant capacity with a similar profile. Moreover, the ORAC assay demonstrated a clear enhancement of the antioxidant content in the *C. officinale* extract compared with the ascorbic acid, as standard.

The stable DPPH radical model is a widely used, relatively quick method for the evaluation of free radical scavenging activity. The effect of plant antioxidants on DPPH radical scavenging is thought to be due to their hydrogen donating ability.[33] The decrease in absorbance of DPPH radical caused by antioxidants because of the reaction between antioxidant molecules and radical, progresses, which results in the scavenging of the radical by hydrogen donation. Table 1 illustrates a significant decrease in the concentration of DPPH radicals due to the scavenging ability of the both **C. officinale** and **L. chuanxiong** extracts and standard. Free radical scavenging activity also increased with increasing concentration. These results indicated that both the extracts have a noticeable effect on scavenging free radicals. The methanol extract of *L. chuanxiong* showed a stronger DPPH scavenging activity than the *C. officinale* methanol extract when compared with standard. We used ascorbic acid as standard.

In addition, the ability to scavenge specific radicals may be targeted. Because different ROS have different reaction mechanisms, to completely determine antioxidant activity against a wide range of ROS, a more comprehensive set of assays needs to be carried out.[32] Superoxide anion (O_2_^·–^) radical is an important factor in biological systems. In order to determine whether inhibition of NBT reduction was due to superoxide scavenger activity, a non-enzymatic system of superoxide generation was used. In the PMS-NADH-NBT system, superoxide anion, derived from dissolved oxygen from the coupling reaction of PMS-NADH, reduces NBT. The decrease in absorbance at 560 nm with antioxidants indicates the consumption of superoxide anion in the reaction mixture. Table 2 shows the percent inhibition of superoxide radical generation by 0–150 µg/ml of **C. officinale** and **L. chuanxiong** methanol extracts compared to that shown by ascorbic acid. **C. officinale** and **L. chuanxiong** methanol extracts showed a dose-dependent inhibition of superoxide radicals. Both the extracts of **C. officinale** and **L. chuanxiong** have strong superoxide radical scavenging activity (IC_50_ = 96.30 and 93.85 µg/ml). Considering the results obtained, it may be anticipated that the methanol extracts of **C. officinale** and **L. chuanxiong** have antioxidant activity, shown here by the scavenging of superoxide radical. IC_50_ values of all these extracts were greater than that of ascorbic acid in which IC_50_ was achieved at 8.76 µg concentration.

**Table 2:** Free radical scavenging and metal chelating activities (IC_50_ µg/ml) of methanol extracts of **C. officinale** and **L. chuanxiong**

	O_2_^·^	H_2_O_2_	OH^·^	NO^·^	Metal chelation
CO	96.259 ± 8.024	136.280 ± 2.307	119.442 ± 7.444	57.252 ± 8.973	138.425 ± 13.292
LC	93.848 ± 9.529	136.318 ± 2.626	113.107 ± 8.890	76.502 ± 3.033^a^*	17.451 ± 5.858^a^*
AA^b^	8.762 ± 4.569	8.053 ± 3.677	3.034 ± 0.191	9.885 ± 0.478	43.235 ± 8.543

Values are means ± SD of three measurements. CO: *C. officinale*, LC: *L. chuanxiong*, AA: Ascorbic acid, O_2_^·–^: superoxide radical, H_2_O_2_: hydrogen peroxide, OH^·^: hydroxyl radical, NO^·^: nitric oxide radical

a Different between CO and LC

* *P* < 0.05 (ANOVA/Tukey)

b positive control

Scavenging of H_2_O_2_ by both the extracts may be attributed to their phenolics, which can donate electrons to H_2_O_2_, thus neutralizing it to water. The H_2_O_2_ scavenging capacities between the two extracts may be attributed to their electron donating abilities.[34] The ability of the both the extracts to effectively scavenge H_2_O_2_ is displayed in Table 2, in which it is compared with that of ascorbic acid as standard. The extracts were capable of scavenging H_2_O_2_ in a concentration-dependent manner. **C. officinale** and **L. chuanxiong** extracts (0–150 µg/ml) exhibited IC_50_ of 136.28 and 136.32 µg/ml, respectively, while ascorbic acid showed 8.05 µg/ml. The correlation between the **C. officinale** and **L. chuanxiong** values was statistically nonsignificant. Although H_2_O_2_ itself is not very reactive, it can sometimes cause cytotoxicity by giving rise to hydroxyl radicals in the cell. Thus, removing H_2_O_2_ is very important throughout food systems.

The **C. officinale** and **L. chuanxiong** methanol extracts were also evaluated for their ability to scavenge hydroxyl radicals using the deoxyribose degradation assay. In this study, the results showed that all samples were able to inhibit deoxyribose degradation (0–150 µg/ml), with a similar profile. The biochemical studies revealed that **C. officinale** and **L. chuanxiong** caused a concentration-dependent inhibition of deoxyribose degradation. At the IC_50_ value level, *C. officinale* (119.44 µg/ml) and *L. chuanxiong* (113.11 µg/ml) exhibited the same potency [Table 2]. Total OH radical scavenging capacities of each extract were compared to that of ascorbic acid.

Plant extracts were measured and compared for their free radical scavenging activities against nitric oxide radicals. The NO^·^ scavenging activity of **C. officinale** and **L. chuanxiong** methanol extracts was examined using SNP as a NO^·^ donor. NO released from SNP reacts with oxygen to produce nitrite. NO scavenger competes with oxygen in reacting with NO^·^ released from SNP solution in PBS. In this study, extracts from **C. officinale** and **L. chuanxiong** showed NO^·^ scavenging capacity [Table 2], although some differences were noted. NO^·^ scavenging activity of *C. officinale* was more significant than *L. chuanxiong*. This inhibition might also be a result of direct scavenging of NO^·^ by extracts. *C. officinale* had the greatest activity to quench NO radical. The IC_50_ values were 57.25 and 76.50 µg/ml for **C. officinale** and **L. chuanxiong**, respectively.

The chelation of ferrous ions by **C. officinale** and **L. chuanxiong** extracts was estimated, in which ferrozine quantitatively forms complexes with Fe^2+^. In the presence of chelating agents, the formation of this complex is disrupted, thereby impeding the formation of the red color imparted by the complex as well. Measurement of this color change therefore allows for the estimation of the chelating activity of the coexisting chelator.[35] In this assay, both the extracts and the standard antioxidant compound interfered with the formation of ferrous–ferrozine complex, suggesting that they have chelating activity, capturing the ferrous ion before it can form a complex with ferrozine. As shown in Table 2, the formation of the Fe^2+^–ferrozine complex is not complete in the presence of the **C. officinale** and **L. chuanxiong** methanol extracts, indicating that both the extracts chelate the iron. The absorbance of Fe^2+^ -ferrozine complex linearly decreased in a dose-dependent manner (0–150 µg/ml). The difference between both the extracts of **C. officinale** and **L. chuanxiong**, and the control was statistically significant. The metal chelating capacities of methanol extracts of **C. officinale** and **L. chuanxiong**, and ascorbic acid (all at IC_50_ µg/ml) were 138.43, 17.45, and 43.24, respectively, which proved to be a significant difference between the extracts and the controls.

**Figure 1:**
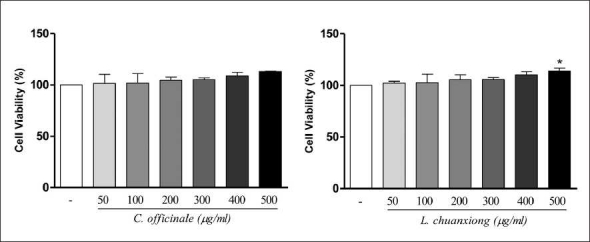
Effect of **C. officinale** and **L. chuanxiong** methanolic extracts on cell viability in N2a cells. Values are means ± SD of three measurements. *P < 0.05 compared with untreated normal (ANOVA/Tukey)

**Figure 2:**
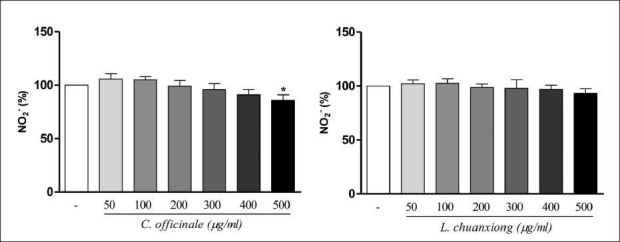
Effect of **C. officinale** and **L. chuanxiong** methanolic extracts on nitric oxide release in N2a cells. Values are means ± SD of three measurements. *P < 0.05 compared with untreated normal. (ANOVA/Tukey)

To determine the effects of **C. officinale** and **L. chuanxiong** on cell viability, the N2a cells were exposed to **C. officinale** and **L. chuanxiong** (50–500 µg/ml) for an incubation time of 1 h. In Figure 1, the MTT test after 1 h of incubation with C. officinale does not indicate any significant viability difference in treated N2a cell cultures in comparison with control. By MTT test after 1 h with *L. chuanxiong*, a significant increase of viability was observed in *L. chuanxiong* 500 µg/ml treated N2a cells in comparison with control. As shown in Figure 2, NO determination was performed after 1 h of incubation in the presence of **C. officinale** and **L. chuanxiong** (50–500 µg/ml). Treatment with *L. chuanxiong* did not decrease the release of NO significantly when compared to control, but 500 µg/ml *C. officinale* decreased significantly the NO release. From this result, it can be concluded that the methanolic extracts of **C. officinale** and **L. chuanxiong**, at the doses used, have no toxicity effects.

## CONCLUSION

Free radical scavenging methods and modifications have been proposed to evaluate antioxidant characteristics and to explain how antioxidant molecules function. Of these, antioxidant activity, free radical scavenging and metal chelation are most commonly used for the evaluation of the total antioxidant behavior of extracts. In the present study, the various free radical scavenging activities of **C. officinale** and **L. chuanxiong** methanol extracts may be attributed to its strong abilities as a hydrogen donor. Moreover, the effectiveness of the methanol extracts of **C. officinale** and **L. chuanxiong** on the cell viability and nitric oxide release in cell culture model has also been established. Results of the present study showed that the selected species of the family Umbelliferae having potential in scavenging of free radicals come out for the therapeutic value.
